# The Enhanced Milk Yield Effect of Early Lactation Increased Milking Frequency and Bovine Somatotropin Is Additive and Not Synergistic

**DOI:** 10.3390/ani13132202

**Published:** 2023-07-05

**Authors:** Haylee H. Hanling, Michael L. McGilliard, Benjamin A. Corl

**Affiliations:** Department of Dairy Science, Virginia Tech, Blacksburg, VA 24061, USA; hayleeh@vt.edu (H.H.H.);

**Keywords:** milking frequency, somatotropin

## Abstract

**Simple Summary:**

Temporarily increasing milking frequency, the number of milkings per day, from twice daily to four times daily for three weeks postpartum, enhances milk yield throughout lactation. Another management practice to increase milk production is the administration of bovine somatotropin, a naturally occurring growth hormone in cattle. In this study, cows underwent increased milking frequency in early lactation and received bovine somatotropin at mid-lactation to determine if the milk yield stimulating effects would act synergistically. In combination, these methods enhanced milk yield additively but not synergistically.

**Abstract:**

Dairy farm profitability depends on milk yield, so the dairy industry manages cows to improve their productivity. Both bovine somatotropin (bST) and early lactation increased milking frequency (IMF) and milk yield (MY) in dairy cows. The objective of this study was to evaluate the effects of mid-lactation bST administration on milk production in established lactation when combined with the milk yield carry-over effect from early lactation IMF. Thirteen multiparous Holstein cows were milked unilaterally for 20 days in early lactation. The left udder halves were milked twice daily (2X) and the right udder halves were milked four times daily (4X). Udder halves milked 4X produced 8.60 ± 1.40 kg more than 2X on the final day of IMF treatment. Cows were then returned to 2X milking for the remainder of lactation and sampled on alternate days from 74–94 days in milk (DIM). Bovine somatotropin was administered to all cows at 80 DIM. The 4X halves continued to make 2.66 ± 0.12 kg/d more milk than 2X through 94 DIM. Fat, protein, and lactose yields were significantly greater in the 4X halves compared to the 2X from 74–94 DIM. Overall milk yield increased by 2.71 kg/d with bST administration. However, there was no significant interaction between MF and bST administration. We can infer from these data that the mechanisms by which bST and IMF in early lactation increase milk yield are complementary due to their non-synergistic nature of enhancing MY.

## 1. Introduction

Milk yield is a key determinant of dairy farm profitability and management approaches influence milk yield. Bovine somatotropin (bST) is a naturally occurring hormone in cattle that can increase milk yield (MY) and improve lactation persistency. When recombinant bST is administered to dairy cows, it increases MY by 15–20% during the course of its effectiveness [1,2,3,4]. The injection is administered starting between wk 9 and 10 of lactation and then every 14 days for the remainder of lactation; this increases the overall milk yield through lactation by 4.5 kg/d [5]. Milking frequency is another management factor known to influence MY, and early lactation increased milking frequency is a specific approach that affects milk yield beyond the application period of increased milking frequency. Research has shown that cows milked four times daily (4X), as opposed to twice daily (2X), for the first 21 days of post-calving had a persistently increased MY in the 3 wk time frame and throughout lactation when returned to 2X milking. During the IMF period, cows produced 5 kg/d more milk than those milked 2X [6,7].

Milk yield is dependent on the number and activity of secretory mammary epithelial cells [8]. Factors that influence milk yield alter one or both. The biological mechanism behind the increased MY carry-over effect, due to IMF in early lactation, is presumed to be due to the increased cell number [9], although the effect on mammary epithelial cell proliferation has not been detected consistently [10]. Administration of bST has also been demonstrated to increase the proportion of proliferating cells [11] and aid in the maintenance of mammary epithelial cell numbers [12]. Bovine somatotropin also increases circulating nutrient availability for milk production to support increased milk production by mobilizing adipose tissue and decreasing the oxidative metabolism of glucose in the muscle. Glucose metabolism is influenced by bST through an increased rate of hepatic gluconeogenesis and insulin insensitivity, which reduces the overall use of glucose by non-mammary tissues. This increases blood glucose availability for the mammary gland [13].

Previous studies combined IMF and bST simultaneously to examine the milk yield effects in established lactation. Knight et al. [14] milked mid-lactation primiparous cows unilaterally with 2X and 4X contralateral udder halves with or without bST. Both bST and IMF alone increased MY, and the combination of treatments further enhanced the yield [15]. Increased MY beyond treatment was largely due to IMF rather than bST. Increased milking frequency also increased epithelial cell size independent of bST. The study concluded that bST and mid-lactation IMF utilized independent mechanisms to increase the MY, which were additive but not synergistic in nature.

Vanbaale et al. [16] combined early lactation IMF and bST in cows either milked three (3X) or six (6X) times daily with bST administered in mid-lactation. Cows milked 6X in early lactation were returned to 3X milking for the remainder of lactation. The 6X cows that received bST produced significantly more milk than the 6X control cows. However, the 3X control cows produced more milk than all 6X cows for the first 9 wks of lactation. The researchers attributed this to increased standing time and reduced resting and feeding time. Therefore, the interaction of bST and early lactation IMF was not adequately evaluated.

The objective of this experiment was to examine the acute milk yield stimulatory effect of bST administration in established lactation, combined with the carry-over milk yield effect of early lactation IMF thought to be a result of increased mammary cell numbers. To do so, we monitored udder half MY differences (4X-2X) in mid-lactation before and after bST injection. We hypothesized that IMF in early lactation, combined with bST in mid-lactation, would function synergistically to further enhance the udder half difference in MY between the 2X and 4X udder halves compared to IMF treatment without bST.

## 2. Materials and Methods

### 2.1. Animal Sampling

All animal procedures were approved by the Virginia Tech Institutional Animal Care and Use Committee (17-001). Cows were housed at Virginia Polytechnic Institute and State University’s Kentland Farm. Thirteen multiparous Holstein cows with an average lactation number of 2.85 ± 0.99 underwent unilateral frequent milking (UFM) for 20 days in early lactation; the left udder halves were milked 2X and the right 4X. Udder half difference was measured at 3 days in milk (DIM) to ensure equal production. Animals were added to the experiment if their udder half milk weights had less than a 0.75 kg difference at a 6 h milking interval and were free of clinical mastitis. The whole udder was milked at 0100 and 1300 and the right halves were milked additionally at 0700 and 1900. Cows were sampled for milk yield and components at the end of the 4X treatment period using a Surge RX quarter milker, which collected milk from each udder half into separate buckets. The animals were returned to normal 2X milking the following day. Milk samples were analyzed by DHIA (Lancaster, PA, USA) for fat, protein, and lactose percent using Fourier-transform infrared spectroscopy (Foss Milkoscan FT+, Foss North America, Eden Prairie, MN, USA). Component percentages were multiplied by milk weight to obtain component yields. The somatic cell count was measured using flow cytometry (Foss Fossomatic FC, Foss North America).

The cows were sampled again during mid-lactation every other day from 74–94 DIM using a quarter milker to separate udder milk yield by udder half. Cows received a single subcutaneous injection of recombinant bST on d 80 (Posilac, Elanco, Greenfield, IN, USA). Udder half difference of milk and component yields before bST injection (d 74, 76, 78, and 80) provided the increased MY carry-over effect due to 4X milking. Sampling days after d 80 indicated the treatment effect of bST on udder half difference in yields.

### 2.2. Statistical Analysis

Sample size was calculated from a power analysis using Statistical Analysis Software (SAS, Cary, NC, USA, version 9.4) with data from previous experiments that showed significant differences in udder half MY due to 2X and 4X MF in early lactation. The analysis indicated the need for 12 animals to achieve a power of 0.8, and 14 cows were enrolled in the study to account for unforeseen animal losses. One cow was euthanized on the 19th day of 4X milking due to peritonitis. Statistical analyses were performed using SAS and the GLIMMIX procedure for udder half milk, fat, protein, and lactose yields as well as somatic cell scores (SCS). Somatic cell score was natural log transformed to achieve normality. Samplings represented individual milking, so yields were doubled to represent yield per day. Samples from the final day of 4X treatment (23 DIM) were analyzed with MF as a fixed effect and cow as a random effect. Fixed effects for the analysis in mid-lactation (74–94 DIM) were treatment, MF, treatment by MF interaction, and day nested within treatment. Cow, cow by MF interaction, and cow by MF by treatment interaction were included as random effects. Additionally, the random effect of the interaction between cow and treatment was the subject of treatment nested within a day. The effect of MF and treatment by MF interaction were tested by the cow by MF interaction. Treatment was defined as either control (74–80 DIM) or bST (82–94 DIM). Sampling days on 74, 76, 78, 80, 82, 84, 86, 88, 90, 92, and 94 DIM signified day, and MF was either the 2X or 4X udder half yields. Increased MF was not reapplied in mid-lactation, but samples were still collected by udder half (either 2X or 4X) to evaluate the carry-over effect of IMF in early lactation. Treatment (control or bST) was evaluated as a repeated measure because all cows received bST and were sampled multiple times before and after administration. Udder half differences existed if there was a significant difference between the 2X and 4X treatments. A Tukey analysis was used to identify significant differences between mean udder half yields at 23 DIM (the final day of IMF), 74–80 DIM (the IMF carry-over effect period), and 82–94 DIM (the IMF carry-over effect period with bST). Two 4X udder half observations were excluded from analysis, one for protein and another for SCC, because studentized residuals were 4.8 and 4.6, respectively. A multiple comparisons test adjusted to Bonferroni was used to compare the differences in means before and after bST administration. The milk, protein, and lactose yield data of one cow on d 74 and another on d 84 were excluded for being off feed. To evaluate the relationships among MY differences due to IMF and bST, simple linear regression was computed using the Solution option within the model statement of GLIMMIX in SAS. Models evaluated the relationship of udder half difference after early lactation IMF and in mid-lactation, as well as udder half MY differences before and after bST administration. A third model evaluated the relationship between the mid-lactation udder half milk yield difference and the milk yield effect of bST administration (difference of post- and pre-bST). The results are presented as the mean ± standard error. Significance was defined as *p* ≤ 0.05 and the tendencies were set to *p* ≤ 0.10.

## 3. Results

Early lactation IMF was highly successful in increasing MY in this study. The 4X udder halves produced 8.60 ± 1.07 kg/d more milk than the 2X udder halves on the 20th and final day of 4X milking, a 38% increase in milk yield (*p* < 0.05; Table 1). The IMF glands also produced more fat, protein, and lactose compared to the 2X on the final day of 4X milking (*p* < 0.01; Table 1). The udder half difference (4X-2X) of these yields on the final day of IMF was greater than all mid-lactation sampling days for milk (*p* < 0.001; Table 2), fat, protein, and lactose yields.

**Figure 1:**
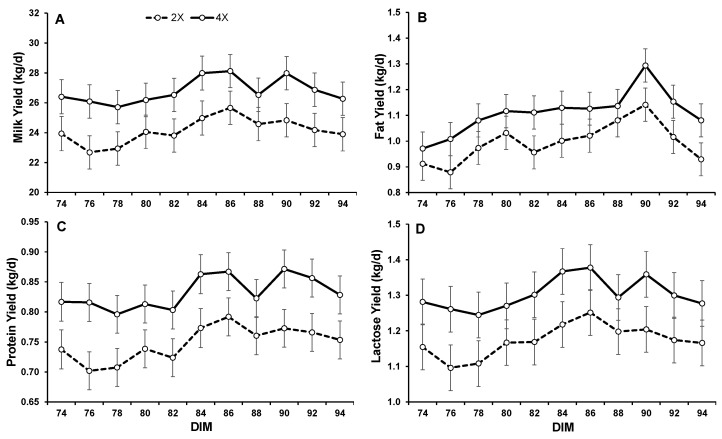
Milk and milk component yields from d 74–94 of cows milked 2X and 4X in left and right udder halves for 20 days in early lactation. (**A**) Milk yield; (**B**) milk fat yield; (**C**) milk protein yield; and (**D**) milk lactose yield. Days 74–80 are control sampling days before administration of bST, and d 82–94 represent the bST treatment period. Bovine somatotropin was administered after milking at 80 DIM. Data are presented as least squares means ± SEM (n = 13).

**Figure 2:**
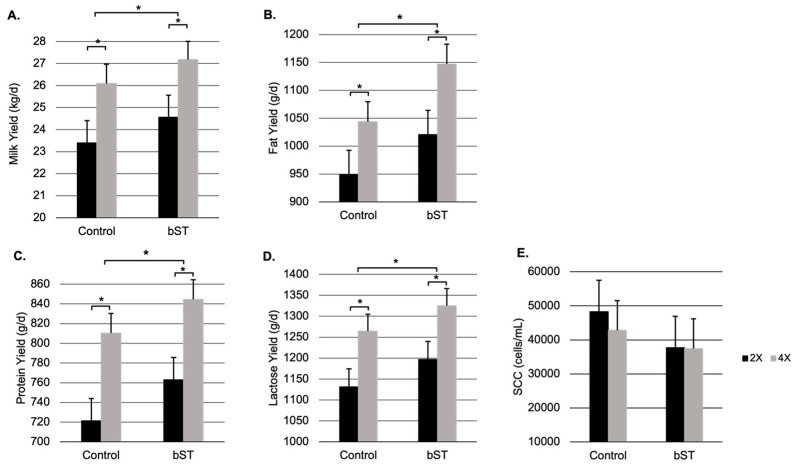
Comparison of milk and component yields between milking frequency and bST treatment. (**A**) Milk yield; (**B**) milk fat yield; (**C**) milk protein yield; (**D**) milk lactose yield; and (**E**) somatic cell count. Control bars represent 74–80 DIM, and bST bars represent 82–94 DIM. Bovine somatotropin was administered after milking at 80 DIM. Black bars indicate 2X and grey bars indicate 4X milking frequencies. Data are expressed as least squares means ± SEM (n = 13). * = *p* < 0.05 for comparisons of 2X and 4X as well as pre and post-bST administration.

An increased MY, due to 4X milking, carried over through 94 DIM. Milk production by the 4X glands was 2.7 kg/d and 11% greater than the 2X (Table 2, Figure 1). Increased milking frequency also increased fat, protein, and lactose yields from 74–94 DIM (*p* < 0.0001; Table 3; Figure 1 and Figure 2). The somatic cell scores were slightly lower in the 4X halves compared to 2X (*p* = 0.09; Table 3; Figure 2). However, apart from two chronically subclinical outliers, all SCCs were below <100,000 cells/mL.

Milk yield responded positively to the administration of bST (*p* = 0.04; Table 4). Though total MY increased by 2.71 kg/d after bST injection, the interaction between IMF and bST treatments was not significant (Figure 1). Both bST and IMF increased milk production but did not have a combined effect greater than the combination of their individual effects. Therefore, the two methods of increasing MY functioned additively but not synergistically. Bovine somatotropin also increased fat, protein, and lactose yield in both the 2X and 4X halves (*p* < 0.05; Table 4; Figure 2). There was no interaction between bST and IMF to further increase component yields.

We compared the 2X and 4X udder half MY between early lactation, mid-lactation, and mid-lactation after bST administration (23, 74–80, and 82–94 DIM, respectively; Table 2). Udder half MY from the 4X right udder halves on the final day of IMF treatment (23 DIM) was greater than yields from right udder halves in mid-lactation pre- and post-bST injection (*p* < 0.05). There was no difference between the 4X MY pre- and post-bST administration, but bST caused a significant increase in MY on the whole udder level. There was also no difference in MY between the 2X udder halves in early lactation, mid-lactation, or mid-lactation post-bST injection. Interestingly, MY from the 4X glands pre-bST and the 2X glands post-bST were similar.

**Figure 3:**
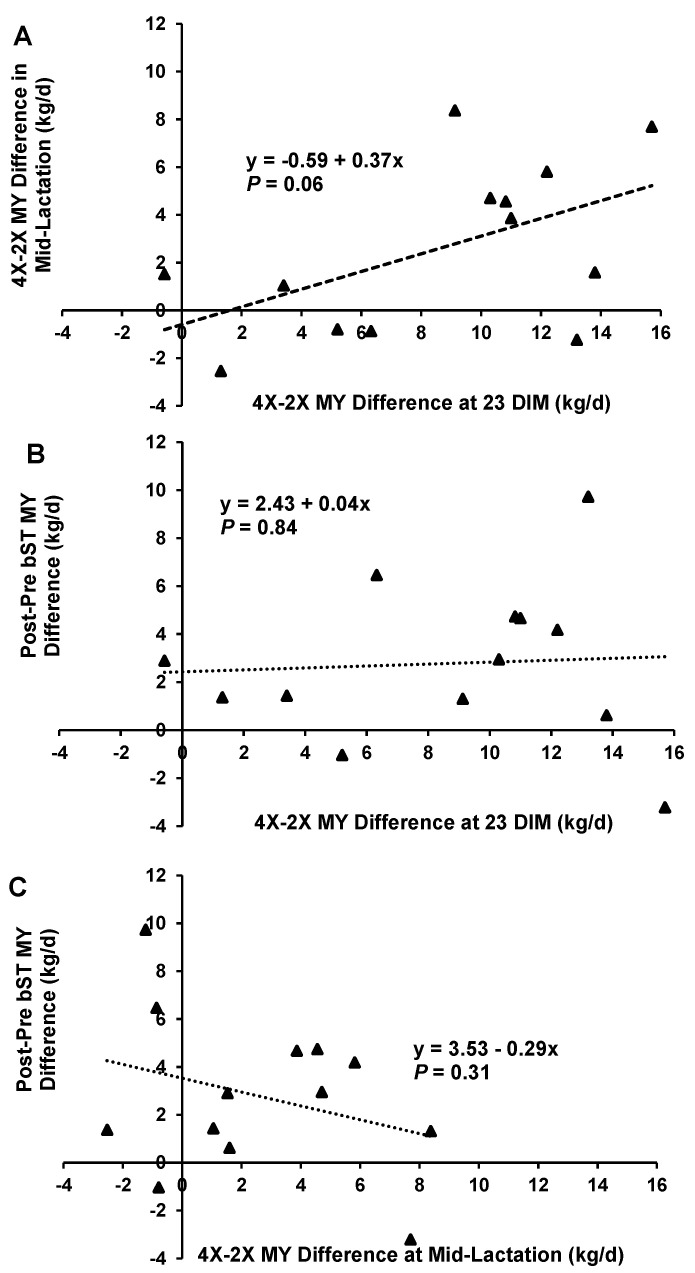
Scatter plots that correlate responsiveness to increased milking frequency in early lactation, in mid-lactation, and to bST administration. Each triangular point represents one cow in the study. Responsiveness to IMF in early lactation was calculated by subtracting 4X-2X udder half milk yields. Bovine somatotropin was administered at 80 DIM. The responsiveness to bST was calculated by the average whole udder milk yield post-injection (82–94 DIM) minus the average whole udder milk yield pre-injection (74–80 DIM). (**A**) There was a tendency for responsiveness in early lactation to correlate to an IMF treatment effect in mid-lactation; (**B**) there was no correlation between responsiveness to IMF at 23 DIM; or (**C**) the average 4X-2X milk yields from 74–94 DIM.

There was variation in the response of cows to IMF. Most cows responded positively to the increased milking frequency at the end of the early lactation IMF period, but not every cow responded to IMF. Responses ranged from less production in the 4X half than the 2X half to more than 10 kg/d on a whole udder basis (Figure 3A). There was a tendency for cows that responded to IMF at 23 DIM to have an increased MY carry-over effect from 74–94 DIM (*p* = 0.06; Figure 3A). Cows consistently responded to bST administration with the exception of two, but there was also variation among cows in response with a range of 12 kg/d (Figure 3B). There was no relationship between responsiveness to early lactation IMF at 23 DIM and responsiveness to bST (Figure 3B), and there was also no relationship between responsiveness to IMF from 74–94 DIM and responsiveness to bST (Figure 3C).

## 4. Discussion

Increased MF in early lactation causes a carry-over effect where the MY continues to be enhanced throughout lactation, although cows have returned to routine MF. This study examined the potential for synergy between the carry-over effect of IMF in early lactation and its interaction with bST. Both bST use a 4X MF increased MY in mid-lactation. However, there was no synergistic effect when combining the two treatments; the improved MY effect of 4X MF was the same before and after bST administration.

There was no synergistic effect between bST administration and IMF when implemented simultaneously in an experiment utilizing primiparous cows [14]. First lactation cows were unilaterally milked with 2X and 4X contralateral udder halves in mid-lactation with or without bST. Both bST and IMF alone increased MY, and the combination of treatments further enhanced yield, meaning they functioned additively [14]. However, bovine somatotropin did not further enhance the increased MY effect of IMF, so the two treatments did not function synergistically. There are some similarities between our study and Knight, Hillerton, Kerr, Teverson, Turvey, and Wilde [14], but a key difference is the lactation stage. We implemented IMF in early lactation and administered bST in mid-lactation; whereas Knight et al. [14] implemented both practices in mid-lactation. Those researchers observed that an increased MY persisted beyond treatment, and this effect was largely due to IMF rather than bST. Both the 4X + bST group and the group that received 4X milking alone showed an increased MY carry-over effect after the 14 d efficacy of bST compared to the 2X groups with or without bST. We chose to implement IMF in early lactation to cause changes in the mammary gland which resulted in the milk yield carry-over effect and examine the effect of the stimulatory effect of bST on milk yield in these modified mammary glands. There is little evidence that this effect occurs at any other lactation stage. However, we administered bST in mid-lactation because it does not increase MY in early lactation when cows are in a negative energy balance [17].

Lactating goats have been used to evaluate the interaction of somatotropin and milking frequency either concurrently or independently in established lactation. Unilateral IMF in established lactation did not produce a milk yield carry-over effect from the 3X to the subsequent 2X period, and bST administration in the subsequent 2X period produced similar increases in milk yield between udder halves [15]. When bST was applied concurrently with unilateral 1X and 3X milking, bST increased the milk yield in 3X udder halves more than the 3X control udder halves and the interaction of MF and bST on MY was not significant [18]. Our study induced changes in the mammary gland due to the application of IMF in early lactation which persisted into established lactation that could have induced differential MY responses following bST application. The early lactation application of IMF contrasts with the studies of Knight [15] and Boutinaud, Rousseau, Keisler, and Jammes [18], but whether increased milking frequency was applied in early or established lactation, concurrently or independently of bST administration, there were no interactions of bST and increased milking frequency. The effects were simply additive.

To our knowledge, the 8.60 ± 1.07 kg/d increase in MY observed in this study by the 4X udder halves compared to the 2X on the last day of treatment at the end of IMF treatment in early lactation is the largest response in the reported literature. The increased MY effect persisted through 94 DIM with the 4X halves producing 2.37 kg/d more milk than the 2X halves. In a previous study, our lab observed a 6 kg/d increase in MY in the 4X compared to the 2X udder halves at 25 DIM after the 21 d UFM treatment period [19]. The increased MY carry-over effect averaged 1.6 kg/d through 300 DIM. Primiparous cows did not have a significantly enhanced MY carry-over effect but multiparous cows responded to the 4X treatment as expected. Therefore, we chose to only utilize multiparous cows for the present study. The exclusion of primiparous cows likely explains the greater response to early lactation IMF.

The better response of multiparous cows to early lactation IMF is supported by previous literature. Wright et al. [20] utilized 4X and 2X UFM for 21 days in early lactation in 16 primiparous cows. They found an MY increase of 2.8 kg/d in the 4X udder halves compared to the 2X at the end of the IMF period, corresponding to a 6.25% increase in energy-corrected milk. When the cows were returned to 2X milking for the whole udder, the average MY increase was 0.8 kg/d in the 4X udder halves through 270 DIM [20]. This increased MY carry-over in the IMF glands corresponded to a 6% increase in energy-corrected milk. The same lab utilized similar methods in six multiparous cows. A key difference was that these multiparous cows had a 6.6 kg/d, or a 33% MY increase in the 4X udder halves [7]. The cows were sampled through 180 DIM, and the increased MY effect persisted in the 4X halves by 2.7 kg/d.

Bauman et al. [5] estimated the MY responses to bST could be up to 5 kg/d and a more recent meta-analysis indicated that MY is increased by 4 kg/d by bST [21]. However, the average response observed by Bauman, Everett, Weiland, and Collier [5] in the study across lactation (post 60 DIM) was 2.9 kg/d, and multiparous cows had a lower response than primiparous. The increase in MY, due to bST, was lower in early and peak lactation until approximately 130 DIM. The response plateaued through mid- and late lactation at 3.6 kg/d [5]. Other reports have shown increases in the order, ranging from 2 kg/d up to almost 6 kg/d for multiparous cows [2,3,4]. The multiparous peak lactation cows in the present study had a similar average response of 2.7 kg/d on the whole udder level to the bST treatment.

Milk yield is dependent on secretory mammary epithelial number and activity [8,22]. Increases in milk yield in response to bST administration and IMF can be traced to increases in mammary epithelial cell number [9,11,12]. In the case of IMF, effects on the mammary gland are locally mediated [8,23]. Bovine somatotropin functions systemically, partially by enhancing nutrient availability to support an increased milk synthesis [13]. The enhanced nutrient availability, caused by bST, supplies the mammary gland with greater amounts of nutrients like glucose, amino acids, and fatty acids to create milk components. The induction of increases in milk yield is associated with IGF-1 synthesized in the liver to act on mammary epithelial cells. Cows that received daily injections of bST for 12 days showed a 3-fold increase in serum IGF-1 after 2 days [24]. A local arterial infusion of IGF-1 in goats was also able to increase milk yield acutely, although the response was blunted by IMF [25,26]. The acute stimulatory effect of bST on milk yield, whether via IGF-1 acting on mammary epithelial cells, as shown in some studies, or through another mechanism facilitated by the homeorhetic effect of bST on nutrient metabolism, did not act synergistically in this study upon the increased mammary epithelial cell number present as a result of early lactation IMF.

## 5. Conclusions

In conclusion, early lactation IMF significantly increased milk, fat, protein, and lactose yields in the 4X udder halves compared to the 2X through 94 DIM. Bovine somatotropin administration at 80 DIM significantly increased milk and lactose yields and tended to increase fat and protein yields throughout its 14 d efficacy. However, bST did not alter the increased milk and component yield effect of early lactation IMF. Udder halves milked more frequently in early lactation produced similarly enhanced milk, fat, protein, and lactose yields before and after bST administration.

## Figures and Tables

**Table 1:** Milk and milk component yields by udder half (either 2X or 4X milking frequency) on the final day of 4X milking treatment (23 DIM).

	2X	4X	SEM	*p*-Value ^1^
Milk Yield (kg/d)	22.83	31.43	1.25	<0.0001
ECM (kg/d)	26.43	36.87	1.55	0.0004
4% FCM (kg/d)	25.11	35.02	1.61	0.001
Fat Yield (g/d)	1065	1496	93	0.0067
Protein Yield (g/d)	676	944	31	<0.0001
Lactose Yield (g/d)	1090	1540	65	<0.0001
ln(SCC)	5.7	4.6	5.6	0.10

^1^ *p*-values shown are between 2X and 4X yields. Significance was declared at *p* < 0.05. Tendencies were declared at *p* ≤ 0.10.

**Table 2:** Milk yields (kg) by udder half (either 2X or 4X milking frequency) in kg/d on the final day of early lactation IMF (23 DIM), mid-lactation prior to bST injection (every other day from 74–80 DIM), and after bST injection (every other day from 82–94 DIM). SED represents the standard error of the difference in means.

	2X	4X	SED
End of IMF	22.8 ^d^	31.4 ^a^	1.75
Pre-bST	23.0 ^d^	25.7 ^bc^	1.02
Post-bST	24.4 ^cd^	27.0 ^b^	1.22

^a–d^ Means without a common superscript differ between 2X and 4X milking frequency treatment across lactation stages (*p* < 0.05).

**Table 3:** Milk and milk component yields by udder half (either 2X or 4X milking frequency). Yields are the average of mid-lactation sampling 74–94 DIM with bST administration on d 80 of lactation.

	2X	4X	SEM	*p*-Value ^1^
Milk Yield (kg/d)	24.0	26.6	0.8	0.02
ECM (kg/d)	26.43	29.34	0.83	0.02
4% FCM (kg/d)	24.55	27.23	0.82	0.02
Fat Yield (g/d)	985	1096	30	0.02
Protein Yield (g/d)	742	827	18	0.02
Lactose Yield (g/d)	1164	1295	39	0.03
SCS	4.0	3.9	0.4	0.51

^1^ *p*-values shown are between 2X and 4X yields. Significance was declared at *p* < 0.05.

**Table 4:** Effect of bST on milk and component yields. Values represent mean and standard error of the difference in means (SED). Control values are mean udder half yields from 74–80 DIM, and bST values are mean udder half yields from 82–94 DIM.

	Control	bST	SED	*p*-Value ^1^
Milk Yield (kg/d)	24.7	25.9	0.55	0.04
Fat Yield (g/d)	1010	1094	38	0.03
Protein Yield (g/d)	766	804	18	0.02
Lactose Yield (g/d)	1195	1261	27	0.02
ECM (kg/d)	26.99	28.78	0.73	0.02
4% FCM (kg/d)	25.02	26.76	0.74	0.03
SCS	4.0	3.9	0.15	0.61

^1^ *p*-values shown are between Control and bST yields. Significance was declared at *p* < 0.05.

## Data Availability

The data presented in this study are available on request from the corresponding author.
